# Mini-dose methotrexate combined with methylprednisolone as a first-line treatment for acute graft-versus-host disease: A phase 2 trial

**DOI:** 10.2478/jtim-2023-0111

**Published:** 2023-09-02

**Authors:** Zhengli Xu, Xiaodong Mo, Yuan Kong, Qi Wen, Tingting Han, Meng Lyu, Lanping Xu, Yingjun Chang, Xiaohui Zhang, Xiaojun Huang, Yu Wang

**Affiliations:** Peking University People’s Hospital, Peking University Institute of Hematology, National Clinical Research Center for Hematologic Disease, Beijing Key Laboratory of Hematopoietic Stem Cell Transplantation, Collaborative Innovation Center of Hematology, Peking University, Beijing 100044, China; Peking-Tsinghua Center for Life Sciences, Academy for Advanced Interdisciplinary Studies, Peking University, Beijing 100044, China

**Keywords:** allogeneic hematopoietic stem cell transplantation, graft-versus-host disease, T cells, methotrexate

## Abstract

**Background and Objectives:**

Acute graft-versus-host disease (aGvHD) remains a major complication after allogeneic hematopoietic stem cell transplantation (allo-HSCT). Methylprednisolone (MP; 1–2 mg/kg/day) remains the standard first-line therapy for aGvHD, although no response is detected in nearly one-half of the patients with aGvHD. This study aimed to investigate the feasibility of mini-dose methotrexate (MTX) combined with standard-dose MP as a front-line therapy for aGvHD.

**Materials and Methods:**

A prospective Phase 2 clinical trial was performed to evaluate the safety and efficacy of 5 mg/m2 MTX combined with 1 mg/kg/day MP as the initial therapy in 31 patients with aGvHD. Moreover, the effects of MTX combined with MP were explored in a humanized xenogeneic murine model of aGvHD.

**Results:**

The overall response and complete response rate at 7 days after the initial treatment were 100% and 83%, respectively. The overall response rate on day 28 was 87%. The complete response rates for aGvHD grades I, II, and III were 100% (6/6), 82% (18/22), and 66% (2/3), respectively. Grade 3 toxicities occurred in only three patients presenting with cytopenia. Importantly, MTX and MP demonstrated synergistic effects on ameliorating aGvHD in humanized xenogeneic murine model.

**Conclusion:**

The current study suggests that mini-dose MTX combined with standard-dose MP could potentially become a novel first-line therapy for patients with aGvHD.

## Introduction

Acute graft-versus-host disease (aGvHD) is a common complication and a major cause of morbidity and even mortality after allogeneic hematopoietic stem cell transplantation (allo-HSCT).^[1]^ Glucocorticoids (GCs) remain the standard first-line therapy for aGvHD, although no response (NR) is detected in 35%–50% of the patients with aGvHD.^[2, 3, 4, 5, 6]^ The proportion of development to grades III– IV aGvHD and that of requiring secondary graft-versus-host disease (GvHD) therapy with standard-dose methylprednisolone (MP) was reported to be around 20% and 30%–50%,^[7,8]^ respectively. Moreover, no efficacy in response rate was observed in previous reports that combined GCs with other immunosuppressive agents, including mycophenolate mofetil (MMF), antithymocyte globulin (ATG), anti-interleukin-2 receptor antibody, infliximab, or itacitinib, as the first-line therapy for patients with aGvHD.^[3,7–12]^ A meta-analysis of randomized controlled trials (RCTs) comparing various combined front-line therapies with steroid monotherapy even revealed significantly inferior 100-day survival in the combination arm (relative risk = 0.83, *P* = 0.004).^[12]^ Thus, an alternative first-line combination therapy needs to be explored by adding novel forms of immunomodulation against non–immune-related pathways.

In addition to the above-mentioned genetic immunosuppression, other means based on the different pathogeneses of aGvHD may enhance the efficacy of steroids. As a common immunosuppressive agent for GvHD, the effect of methotrexate (MTX) on metabolic checkpoints has been reported. Whether GCs combined with MTX could ameliorate aGvHD and synergistically improve T-cell function needs to be investigated. Our group previously reported that intravenous MTX at a dose of 10 mg or oral MTX at a dose of 15 mg (low dose) combined with a low dose of 0.5 mg/kg/day MP yielded an overall treatment response in 26 out of 32 patients with aGvHD (81%), and the median times to show improvement and achieve a maximal response were 2 and 5 days, respectively, after administration.^[13]^ Although this trial included a prospective cohort, some limitations are present in the pilot study.^[13]^ First, it is difficult to compare the results of 0.5 mg/kg/day MP administration with those of the standard dose of 1–2 mg/kg/day MP used for the first-line treatment of aGvHD.^[1,6]^ Second, the treatment response was evaluated until the maximal response was achieved rather than up to a predefined fixed time point as suggested by standardized terminology and guidance for initial treatment response assessment.^[14]^ Third, the response criteria do not account for secondary GvHD therapy. Considering these limitations, prospective studies with standard-dose MP plus lower-dose MTX and standardized assessment for initial treatment response are needed to challenge systemic steroids as the standard first-line treatment for aGvHD.^[1]^

A reduced dose of 5 mg/m^2^ (mini dose) instead of the standard dose of 10–15 mg/m^2^ MTX was recently used either as prophylaxis or as salvage therapy for aGvHD and showed a higher safety without compromising efficacy.^[15,16]^ Therefore, a prospective Phase 2 clinical trial was performed to evaluate the safety and efficacy of mini-dose MTX (5 mg/m^2^) combined with standard-dose GCs as the first-line therapy in patients with aGvHD. To minimize the development rate to grades III–IV aGvHD and timely initiate secondary GvHD therapy, the primary endpoint was designed to be the overall response rate (ORR) at 7 days after treatment based on the quick response of the previous pilot study with the addition of low-dose MTX to low-dose MP. Moreover, the effects of MTX combined with GCs on activated T cells were explored in a humanized xenogeneic murine model of aGvHD.

## Materials aand methods

### Study design and subjects

The prospective, nonrandomized, open-label study included patients recruited at the Peking University People’s Hospital between December 2020 and May 2021. The study was approved by the ethical committee of Peking University People’s Hospital (2020PHB067-01). All included subjects signed an informed consent. The study protocol was in accordance with the Declaration of Helsinki and was approved by the Institutional Review Board of Peking University. This study was registered at http://clinicaltrials.gov/NCT 04677868.

The study included patients, aged 16–65 years, with aGvHD after hematopoietic stem cell transplantation (HSCT), who had not received drug treatments and whose peripheral blood absolute neutrophil count was higher than 0.5 × 109/L. Exclusion criteria were patients with severe heart, kidney, or liver disease or a life-threatening infection.

A total of 31 patients developing aGvHD following HSCT were screened for inclusion in the trial to receive mini-dose MTX combined with 1 mg/kg MP as an initial therapy for aGvHD. During the study period, 28 patients solely receiving 1 mg/kg MP as a first-line treatment of aGvHD at the physicians’ discretion (owing to grade I acute GvHD, early GvHD onset time, or concerns about risks) were selected as the control cohort. Subject characteristics are shown in Table 1. Human leukocyte antigen (HLA)-A, HLA-B, and HLA-DRB1 typing was performed at the allele level using high-resolution techniques. Two siblings and two unrelated donor– recipient pairs were fully HLA matched.

**Table 1:** Patient characteristics

**Characteristic** Age, years	**MTX group (*n* = 31)** 34 (16–65)	**Control group (*n* = 28)** 27 (16–65)
Male	10	16
Onset of GvHD, days Disease type	22 (11–85)	18 (11–70)
Acute myeloid leukemia	22	15
Acute lymphoid leukemia	2	5
Myelodysplastic syndrome	4	6
Severe aplastic anemia	3	2
Donor and HLA-A, -B, -DR histocompatibility		
Sibling 6/6 matched	2	2
Haploidentical related	27	26
Two HLA antigens mismatched Three HLA antigens mismatched	2 25	2 24
Unrelated 6/6 matched	2	0
Preparative regimen		
Modified BuCy	2	2
Modified BuCy plus ATG	29	26
Grade of acute GvHD		
I	6	13
II	22	7
III	3	8
Site of GvHD		
Skin	25	24
Liver Lower gastrointestinal tract	2 11	1 11
Multiple organs	7	7
Minnesota risk		
standard	29	21
high	2	7
Follow-up from the onset of aGvHD, days	248 (63–352)	248 (47–373)
Follow-up from HSCT, days	270 (106– 241)	270 (63–421)

Data are presented as *n* or median (range). GvHD: graft-versus-host disease; HLA: human leukocyte antigen; MTX: mini-dose methotrexate; ATG: antithymocyte globulin; HSCT: hematopoietic stem cell transplantation; aGvHD: acute graft-versus-host disease; BuCy: Busulfan+Cyclophosphamide.

### Transplant procedures

Conditioning regimen and GvHD prophylaxis have been previously reported in detail.^[17,18]^ All transplant recipients received Busulfan+Cyclophosphamide (BuCy) -b a s ed my e l o a b l a t ive co n d it ion in g regimens.^[19, 20, 21, 22]^ The conditioning regimen included cytarabine (4 g/m^2^/day, day -9), busulfan (3.2 mg/kg/day, intravenously, days -8 to -6), cyclophosphamide (1.8 g/m^2^/day, days –5 to –4), semustine (250 mg/m^2^, day –3), and rabbit ATG (thymoglobulin; Imtix Sangstat, Lyon, France; 2.5 mg/kg/day, days –5 to –2). Cyclosporine A (CsA), MMF, and short-term MTX were administered as GvHD prophylaxis. Haploidentical and unrelated patients received ATG.^[23]^ The dosage of MTX was 15 mg/m^2^, intravenously, on day 1, followed by 10 mg/m^2^ on days 3 and 6 after matched sibling transplantation or on days 3, 6, and 11 after mismatched/haploidentical or unrelated transplantation.

### Study drug administration regimens

Patients received intravenous MTX at a dose of 5 mg/m^2^ and MP at a dose of 1 mg/kg/day. MTX was administered on days 1, 3, 8, and 15 and once in every 7 days afterward. The programmed dose of MP was as follows: days 1–7, 1 mg/kg/day; days 8–14, 0.5 mg/kg/day; days 15–21, 0.25 mg/kg/day, and the dose was reduced by half after 5–7 days until it was stopped. Patients were scheduled to receive at least two doses (number of MTX administrations) for evaluation of the efficacy of the drug. If patients responded and tolerated the toxicity, additional doses were used for consolidation, and the scheduled maximal doses were capped at six.

Patients were observed for 5–7 days and switched to the second-line treatment if there was NR to the initial therapy. The second-line treatment was basiliximab (Novartis Pharma AG, Basel, Switzerland) at 20 mg/day on days 1, 3, 8, and weekly afterward for as long as it was clinically indicated. This second-line treatment was administered to patients for 5 days with progression of aGvHD or for 7 days with no improvement after initial therapy or for 14 days with partial response (PR) after the initial therapy, not including the patients who initially responded to MTX/MP and then flared.^[14]^

### Definition and evaluation

Before treatment initiation, patients underwent a thorough evaluation to ascertain the severity and extent of GvHD, including a physical examination, laboratory evaluations, and a consultation without tissue biopsy results. Each organ (skin, liver, and gut) was staged 1 through 4 for acute GvHD according to the modified criteria based on the schema of the Mount Sinai Acute GvHD International Consortium (MAGIC). Moreover, patients were assigned a grade of acute GvHD (I–IV) based on overall severity. Minnesota GvHD risk status was also evaluated.^[11]^ The time that elapsed between the onset of aGvHD and HSCT was defined as the time from HSCT to the onset of any grade of aGvHD.

A complete response (CR) was classified as complete disappearance of all clinical signs of skin, liver, and/or gut GvHD. It was assumed that a PR occurred if GvHD symptoms in the patient had not completely disappeared, but at least one target organ decreased in grading by at least one stage without deterioration or emergence of GvHD in other organs. Overall responses (ORs) included CR and PR. NR or treatment failure was defined as absence of improvement in any organ involved by aGvHD or worsening in one or more organs by one or more stages, requiring additional systemic GvHD therapy. Meanwhile, changes in white blood cell count and drug side effects were evaluated to assess drug safety. The Common Terminology Criteria (CTC) for adverse events version 3.0 were used to grade the severity of side effects.

### Intracellular flow cytometry

Peripheral blood was harvested from patients before therapy and at 7 and 28 days after therapy. Anti-CD3-APC/H7, anti-CD8- eFluor450, anti-IFN-γ-Percp/Cy5.5, and anti-Ki67 monoclonal antibodies (BD Biosciences, San Jose, CA, USA) were used to stain cell surface markers and intracellular cytokines. CD3+ T cells were cultured with phorbol myristate acetate (100 ng/mL) and ionomycin (2 μg/mL) (both from Sigma-Aldrich, St. Louis, MO, USA) for 4 hours. During this incubation period, GolgiStop (0.7 μL/mL) was added to the samples to sequester cytoplasmic proteins. As previously described, Th1 and Tc1 cells were identified as CD3+CD8−IFN-γ+ and CD3+CD8+IFN-γ+ cells, respectively. Owing to difficulties in obtaining samples, we reserved the peripheral blood of 12 patients who were administered mini-dose MTX combined with 1 mg/kg MP as the initial treatment for aGvHD and six patients who were administered 1 mg/kg MP as the first-line treatment of aGvHD. The samples from MP + MTX group and MP group were matched for age, sex, and time after transplantation.

The proliferation of T cells was examined using a Ki67 antibody (BD Biosciences). First, the cells were harvested and incubated with anti-CD3-APC/H7 antibodies (BD Biosciences) at room temperature (24℃) for 15 minutes. Then, they were fixed and permeabilized with FOXP3 Fix/Perm buffer set (BD Biosciences). Subsequently, the cells were resuspended and incubated with prediluted Ki-67 antibody for 30 minutes on ice in the dark. The proliferation was monitored by the percentage of Ki67-positive cells by flow cytometric analysis.

### Xenogeneic model of aGvHD

G-CSF–mobilized peripheral blood was obtained from healthy donors after they signed a written informed consent approved by our ethical committee. G-CSF–mobilized peripheral blood mononuclear cells (G-PBMC) were harvested from peripheral blood by Ficoll-Paque gradient centrifugation. The current study was approved by the ethical committee of Peking University People’s Hospital (2020PHB067-01).

A humanized xenogeneic aGvHD model was established to further evaluate the effect of MP and MTX on aGvHD, according to a previous report.^[24]^ Six-to eight-week-old NOD.Cg-Prkdc^scid^ Il2rg^tm1Vst^/Vst (NPG; Beijing Vitalstar Biotechnology Co., Ltd., Beijing, China) mice were sublethally irradiated with 1.5 Gy total body irradiation *via* X-ray on day –1, followed by intravenous infusion of 5 × 10^6^ G-PBSCs in the caudal vein, and then were intraperitoneally injected with PBS (CTL), MP (2 mg/kg/day), and MTX (1 mg/kg/day) daily beginning on day 1 after allo-HSCT for 4 weeks. Disease severity was monitored using a scoring system that features six parameters: weight loss, posture, activity, fur ruffling, skin integrity, and diarrhea.^[25]^ Each parameter was assigned a score from 0 (no symptoms) to 2 (severe symptoms), which resulted in a total score from 0 to 12. Tissue samples were prepared, stained with hematoxylin, eosin, and safran, and imaged with microscope (Olympus, Tokyo, Japan) to evaluate GvHD target organ pathology scores.

### Endpoints and statistical methods

The primary endpoint was the ORR to therapy at 7 days after treatment. The study would provide an 80% power with a sample size of 30 patients to detect a 25% benefit in ORR with the mini-dose MTX plus MP, compared to a reference rate of 50% with MP alone that was derived from previous reports. Type I and II error rates were controlled at 5% and 20%, respectively. The secondary endpoints were ORR at 28 days after treatment, incidence of bacterial infections, fungal infections, cytomegalovirus (CMV) and Epstein–Barr virus (EBV) infections, transplant-related mortality (TRM), relapse of original disease, failure-free survival (FFS; defined as alive without relapse, requirement for additional therapy for acute GvHD, or signs or symptoms of moderate-to-severe chronic GvHD), overall survival (OS, time from HSCT to death from any cause), and chronic GvHD incidence.

Data were collected on case report forms by medical record reviews. The time course for aGvHD response and FFS was estimated using the method of Kaplan– Meier. The surviving patients were followed up, and the results of the follow-up examinations were analyzed on December 10, 2021. Unless otherwise specified, all the reported *P* values were based on two-sided hypothesis tests. Statistical Package for the Social Sciences (SPSS) 19.0 (Mathsoft, Seattle, WA, USA) and R version 3.4.4 (The R Foundation for Statistical Computing) were used for data analyses.

## Results

### Patients

Thirty-one patients were enrolled in the study. Most patients were female (68%) and underwent haploidentical HSCT (87%). Median age was 34 (16–65) years. Most of the patients had grade I (19%) or II (71%) aGvHD as per the MAGIC criteria and standard risk (90%) as per the Minnesota risk stratification. Organ involvement was primarily skin (80%), followed by lower gastrointestinal tract (GI) (32%) and liver (6%) (Table 1).

### Responses

aGvHD occurred at a median of 22 (11–85) days after HSCT among the 31 patients in the study group. The drugs were immediately administered after aGvHD was diagnosed. The ORR and CR rate at 7 days after MTX + MP treatment were 100% (31/31 patients) and 83% (26/31), respectively. Among the five patients achieving PR at 7 days after treatment, three achieved CR at 9, 14, and 24 days after MTX + MP treatment without additional second-line treatment and the other two received second-line treatment at 14 and 21 days after MTX + MP therapy. By day 28 after drug administration, among the 29 patients achieving CR after MTX + MP therapy without additional second-line treatment, two flared and received second-line treatment. Therefore, the ORR and CR rate on day 28 were 87% (27/31). Responses in different grades of GvHD (the grades at onset), individual organs involved, and the number of involved organs are shown in Table 2.

**Table 2:** Overall responses and complete responses at 7 days after treatment

**Variable**	**Patients**	**Complete response**	**Overall response**
Total	31	26 (83)	31 (100)
aGvHD grade at onset			
I	6	6 (100)	6 (100)
II	22	18 (82)	22 (100)
III	3	2 (67)	3 (100)
Involved organ			
Skin	25	22 (88)	25 (100)
Stage 1	6	5 (83)	6 (100)
Stage 2	6	4 (67)	6 (100)
Stage 3	13	13 (100)	13 (100)
Gut	11	7 (64)	11 (100)
Stage 1	10	6 (60)	10 (100)
Stage 2	1	1 (100)	1 (100)
Liver	2	1 (50)	2 (100)
Stage 2	2	1 (50)	2 (100)
Multiple organs	7	4 (57)	7 (100)

Data are presented as *n* or *n* (%); aGvHD: acute graft-versus-host disease.

**Figure 1:**
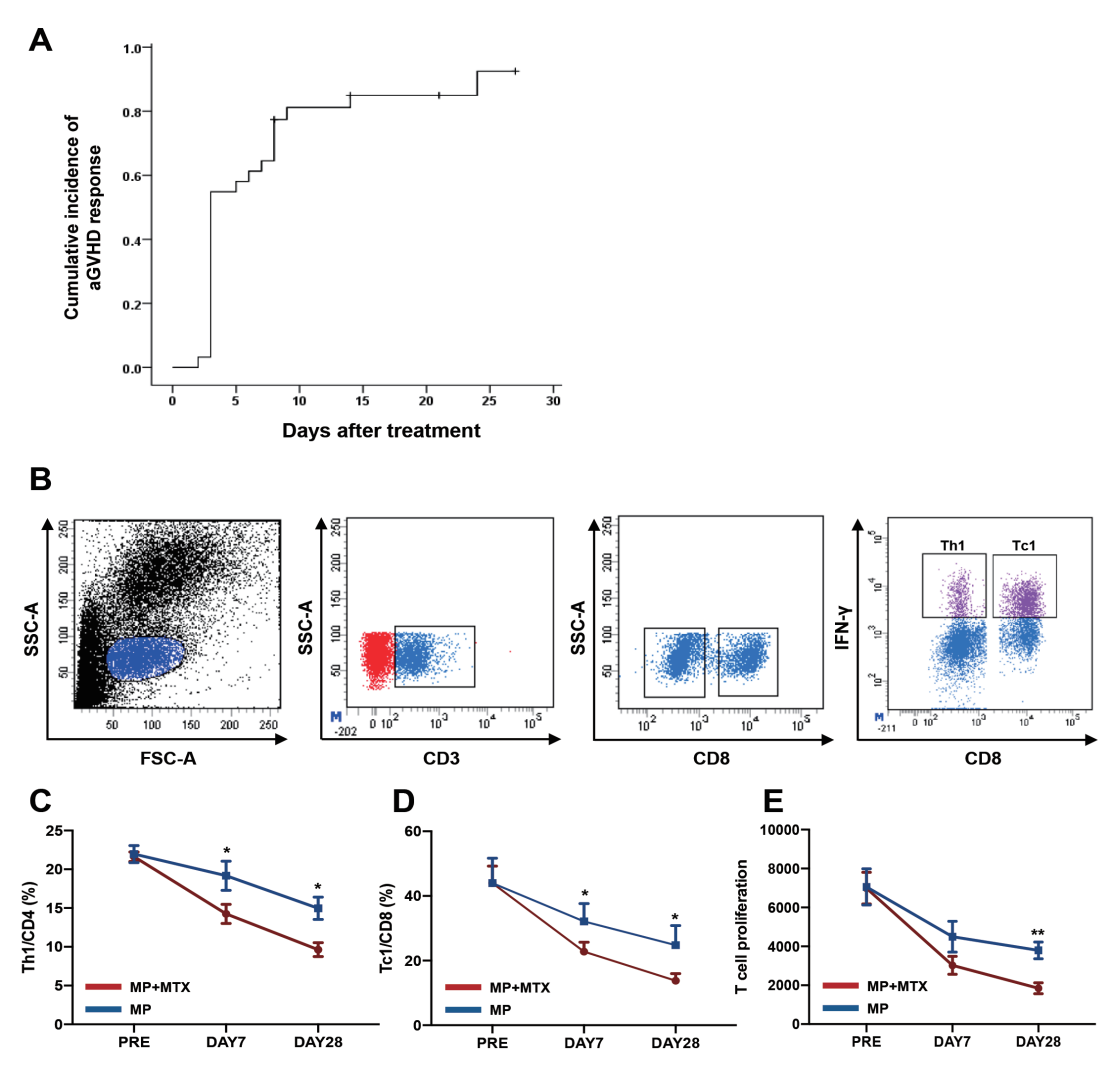
Clinical responses of MP combined with MTX as a first-line therapy in patients with aGvHD. (A) The time course for maximal clinical response of the combination therapy. (B) The representative phenotypes of Th1 and Tc1 cells. Consistent with the clinical responses, the activation, differentiation, and proliferation of T cells in patients were significantly decreased after combined treatment, which was characterized by reduced levels of (C) Th1, (D) Tc1, and (E) T cell proliferation. Data are presented as the means ± SEM (*P ≤ 0.05, **P ≤ 0.01). SSC: side scatter; IFN-γ: interferon γ; Th1: T helper type 1; aGvHD: acute graft-versus-host disease; MP: methylprednisolone; MTX: mini-dose methotrexate; SEM: standard error of the mean.

MTX was administered at a median of 3 (2–4) times. The median time to show improvement (at least reaching PR) for the 31 patients was 3 (2–7) days after application. The median time needed to achieve a maximal response (CR or PR) was 3 (2–24) days. The time course for maximal response is shown in Figure 1A.

In the MP monotherapy group, the ORR and CR rate at 7 days after treatment were 58% (16/28 patients) and 50% (14/28), respectively, and the *P* values were 0.011 and < 0.001, respectively, compared to the combination treatment group. The difference was higher when considering that nearly half of the patients (46%, 13/28) had grade I aGvHD in the MP alone group, whereas the proportion was only 19% (6/31) in the combined treatment group. For grades II–IV aGvHD, the ORR and CR rate at 7 days after treatment were 40% (6/15) *vs*. 100% (25/25, *P* < 0.001) and 33% (5/15) *vs*. 80% (20/25, *P* = 0.006), respectively.

### Immunophenotyping analysis

Consistent with the clinical responses, after treatment with MP and MTX, significantly fewer activated T cells were demonstrated in patients with aGvHD than in those treated with MP alone (Figure 1B–1D), characterized by a reduced proportion of Th1 (Figure 1B; 14.3% ± 1.2% *vs*. 19.2% ± 1.9%; *P* = 0.04) and Tc1 (Figure 1C; 22.8% ± 2.9% *vs*. 32.1% ± 45.5%; *P* = 0.03) cells in patients with aGvHD on day 7 after treatment. Additionally, compared to T cells in patients with aGvHD treated with MP alone, T cells in aGvHD patients treated with a combination of MP and MTX had decreased proliferation (Figure 1D; 3021.7 ± 461.9 *vs*. 4496.0 ± 792.3) on day 7 after treatment. Consistently, the frequencies of proinflammatory cells, such as Th1 (Figure 1B; 9.6% ± 0.9% *vs*. 15.0% ± 1.4%; *P* = 0.01) and Tc1 (Figure 1C; 13.8% ± 2.2% *vs*. 24.8% ± 6.1%; *P* = 0.03) were markedly decreased in the combined treatment group on day 28 after treatment. In addition, degraded T-cell proliferation (Figure 1D; 1846.7 ± 282.8 *vs*. 3792.3 ± 429.2; *P* = 0.003) was observed in the combined treatment group on day 28 after treatment.

### Side effects

Three of the 31 (9.7%) patients developed severe leukopenia (CTC Grade 4, white blood cell < 1 × 109/L) or severe thrombocytopenia (CTC Grade 4, platelets < 25 × 109/L) compared to baseline value after three, one, and two doses of MTX administration, respectively. The white blood cell counts of both the patients developing severe leukopenia were 4.85 × 10^9^ /L and 4.49 × 10^9^ /L before MTX administration, and the nadir values after MTX administration were 0.63 × 109/L and 0.80 × 109/L, respectively. The platelet count of the single patient developing severe thrombocytopenia was 133 × 109/L before MTX administration, and the nadir value after MTX administration was 24 × 109/L. No patients were withdrawn from the study because of hematologic side effects, and their blood cell counts quickly returned to baseline values with a median of 2 days (1–19 days) after the last dose of MTX. In addition, another 12 patients had increased white blood cell and/or platelet counts for at least one lower CTC grade compared to baseline value after MTX administration. Levels of blood glucose and blood pressure remained normal during treatment. During MTX administration, three patients (9.7%) had CMV and/or EBV antigenemia; other infectious complications or nonhematologic toxicities were not observed.

### Follow-up and survival

Up to December 10, 2021, the median follow-up time was 289 (204–421) days from HSCT and 269 days (range: 182–352) from the onset of aGvHD among survivors. Chronic GvHD and moderate-to-severe chronic GvHD occurred in 16 (51.6%) and 6 (19.4%) patients in the MTX group, whereas the incidence was 53.6% (*n* = 15) and 13.4% (*n* = 4) in the control group.

Leukemia relapse occurred in six (19.3%) patients at a median of 103 (82–153) days from HSCT, three of whom eventually died from leukemia relapse. One patient (3.2%) died of lung infection at 154 days from HSCT. Twenty-four patients (77%) remained alive without leukemia relapse with a median survival of 287 (204–421) days from HSCT and 263 (182–352) days from the onset of aGvHD. In the control group, leukemia relapse occurred in two (7.1%) patients at 224 and 282 days from HSCT and four patients (14.3%) died of TRM at a median of 136 (204–421) days from HSCT (two from lung infection and two from GvHD). The 6-month FFS was 61% (95% confidence interval [CI]: 44%–78%) for MTX and 32% (15%–49%) for controls.

### MP combined with MTX demonstrated synergistic effects in humanized aGvHD mice

**Figure 2:**
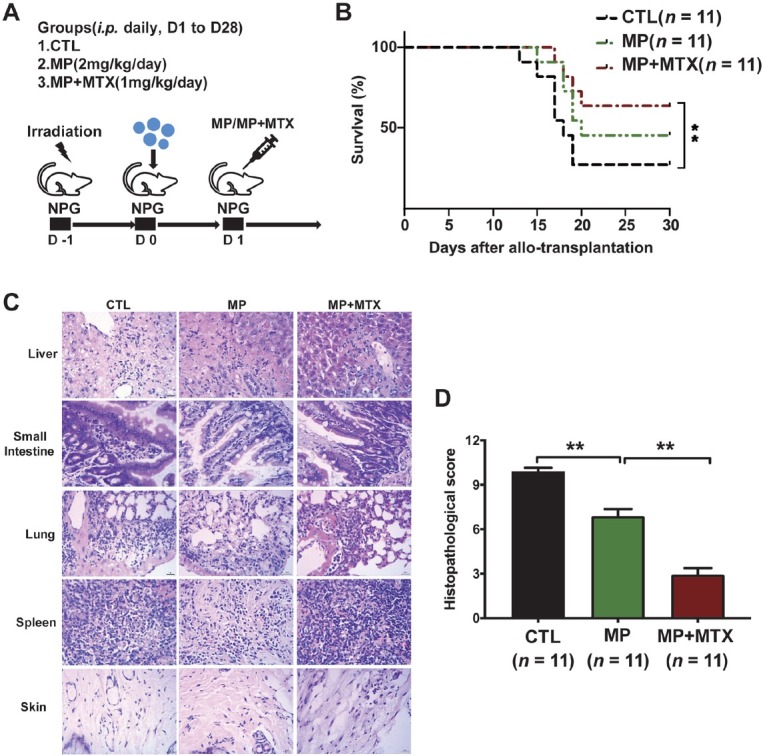
MP combined with MTX synergistically alleviated aGvHD in humanized mice. (A) Protocol for the establishment of the humanized aGvHD mouse model and the different treatment groups. (B) Median survival of the recipient mice in different treatment groups. (C) Representative histology of the target organs harvested from the recipient mice of the different treatment groups. (D) Pathological score of the recipient mice in different treatment groups. Data are presented as the means ± SEM (**P ≤ 0.01). SSC: side scatter; IFN-γ: interferon γ; Th1: T helper type 1; aGvHD: acute graft-versus-host disease. MP: methylprednisolone; MTX: mini-dose methotrexate; CTL: treated with PBS; SEM: standard error of the mean.

To further evaluate the effect of MP and MTX on humanized aGvHD mice model, humanized aGvHD mice were treated with PBS (CTL), MP, and MP and MTX (MP + MTX), respectively. aGvHD occurred in the humanized mice receiving donor G-PBSCs during 1–4 weeks after transplantation, as evidenced by weight loss, disease score (hunching, activity, ruffling, and diarrhea), and death. Similar to patients with aGvHD, humanized aGvHD mice showed severe inflammation, leukocyte infiltration, necrosis, and tissue damage in target organs, such as the skin, lung, liver, and gut, in the G-PBSC group (Figure 2). MP + MTX treatment demonstrated synergistic effects on ameliorating aGvHD, as characterized by improving OS and aGvHD pathological scores in mice compared to those in the CTL or MP alone groups (Figure 2). Consistent with the results of the clinical trial, our data indicated that MP combined with MTX treatment demonstrated better anti-GvHD effect in humanized aGvHD mice model.

## Discussion

In the current prospective Phase 2 trial, our findings indicate that mini-dose MTX combined with standard-dose GCs promises to be a novel first-line therapeutic strategy for patients with aGvHD. GCs combined with MTX demonstrated synergistic effects on reducing T-cell alloreactivity *in vivo* and ameliorating aGvHD in the mice model.

Currently, nearly 50% of the patients with aGvHD have NR to the current standard first-line therapy of 1–2 mg/kg/day MP.^[1,26]^ Moreover, GCs combined with other immunosuppressive agents as the first-line therapy were previously reported to fail in patients with aGvHD regarding response rate and survival,^[3,7–12]^ which suggested the need for alternative combined strategies based on the synergistic mechanisms of immunomodulation. The role of T-cell metabolism in aGvHD has been recently reported, and GCs plus glycolysis inhibitors have been demonstrated to cooperatively abrogate aGvHD in a humanized xenogeneic murine model.^[24]^ MTX is widely used in autoimmune diseases, including aGvHD, to repress T-cell activation and proliferation.^[15,27,28]^ In the current study, we showed that MTX combined with MP substantially alleviated aGvHD in a humanized aGvHD mouse model and demonstrated synergistic effects on ameliorating T-cell activity in patients with aGvHD, confirming the hypothesis of the synergistic effect of MTX and MP. The synergistic benefit of GCs and MTX may be influenced by different mechanisms in clinic, which needs to be further clarified.

In our previous pilot study, intravenous MTX at a dose of 10 mg or oral MTX at a dose of 15 mg (low dose) combined with 0.5 mg/kg/day MP appeared to be an effective regimen when used as a first-line treatment for aGvHD with an ORR of 81% at a median of 5 days after therapy, and the 28-day response rate was 75%.^[13]^ Although the response rate seems higher than that in MP monotherapy reports, it is difficult to be validated in randomized trials that compare the combined therapy by adding agents to a standard dose of 1–2 mg/kg/day MP and MP monotherapy for the first-line treatment of aGvHD. Furthermore, it is postulated that mini**-**dose MTX combined with standard-dose MP might improve the efficacy of the combined strategy. The current results demonstrated increased response rates than those in our previous pilot study in terms of ORR and CR rate in both total cohort (ORR 100% *vs*. 81%, 28-day ORR 87% *vs*. 75%; CR rate 83% *vs*. 75%) and in the subgroups of aGvHD for varying grades (grade I: ORR/CR rate 100% *vs*. 87%; grade II: ORR 100% *vs*. 89%, CR rate 82% *vs*. 72%) and different involved sites (skin: ORR 100% *vs*. 88%, CR rate 100% *vs*. 81%; lower GI: ORR 100% *vs*. 81%; multiple organs: ORR 100% *vs*. 75%).^[13]^ The small number of grades III/IV or liver aGvHD precluded comparisons. In addition, only 6% of the patients in the current study required second-line therapy to achieve a CR, compared to 18% in the previous study.^[13]^ The difference was more remarkable when comparing the current combined treatment arm with the nonrandomized control arm with 1 mg/kg/day MP. A study with a randomized design with a larger sample size comparing this combined strategy and the current standard first-line therapy of 1–2 mg/kg/day MP is ongoing.

Regarding the relationship between the grade of GvHD as well as the donor type and the corresponding treatment, the new information of this study is that a combined therapy of low-grade aGvHD is feasible in patients after haploidentical transplantation. It should be noted that most of the study population had grade I/II aGvHD by the MAGIC criteria or standard-risk GvHD by the Minnesota stratification; thus, they usually respond well to MP. On one hand, the ORR to 1–2 mg/kg MP for grade I/II aGvHD was shown to be 60%–66%,^[29,30]^ whereas the proportion of standard-risk GvHD by the Minnesota criteria was 85% among all transplant patients or 77% among patients with higher than grade II aGvHD, and the ORR to 2 mg/kg MP for standard-risk aGvHD was 68%–70% in previous reports.^[9,31]^ In other words, the current combined strategy may further improve the response rate for this kind of patients, especially in haploidentical setting. On the other hand, as per the EBMT guideline, grade I cases could have been treated with topical steroids alone.^[1]^ However, in a randomized trial, although steroid treatment of acute grade I GvHD prevented progression to grade II but not to grade III–IV GvHD, it was found that an early onset of GvHD was a substantial negative predictor of survival, independent of the randomization arm.^[32]^ In the Minnesota risk stratification study, days from HCT to aGvHD (< 28 *vs*. > 28 days) also substantially affected TRM and survival.^[31]^ Considering the median aGvHD onset time of 22 days in the current population, mainly in haplo-setting, MP treatment may be initiated for grade I aGvHD after haplo-HSCT, as recommended by the Chinese consensus on GvHD. ^[33]^ A Phase 2 trial of 2 mg/kg/day MP combined with mini-dose MTX for higher grade aGvHD is underway. Although the response was usually determined on day 14 (±7 days), 28 (±7 days), and 56 (±14 days) after prednisone treatment was initiated,^[7,8,10,11,14,25,29,31,34]^ the ORR on day 7 was chosen as the primary endpoint according to the quick response with low-dose MTX and low-dose MP, which is in line with the result obtained from the trial of combined therapy of infliximab with steroids.^[8]^ Moreover, timely secondary therapy can be administered to steroid-refractory patients with earlier judgment and may preclude GvHD development.

The rate of developing severe cytopenia during therapy in this trial is similar to that in the previous pilot study (9%)^[13]^ and seemed to be lower than those in previous reports on MTX treatment for GvHD without MP,^[35,36]^ possibly owing to the “mini”-dose of MTX and the combination strategy used. Shiratori *et al.*^[15]^ recently reported that GvHD prophylaxis using mini-dose of MTX 5 mg/m^2^ instead of 10–15 mg/m^2^ in cord blood transplantation (CBT) is associated with improvement of engraftment and reduction in nonrelapse mortality (NRM). Besides, MP might partly mitigate cytopenia caused by MTX. Nevertheless, close monitoring of blood cell counts is necessary whenever MTX is administered. No other commonly documented side effects, such as gastrointestinal symptoms, renal dysfunction, or immune-mediated pneumonitis, were observed. The occurrence of CMV/EBV antigenemia during the study drug administration was similar between the current report and the previous pilot study (9%).^[13]^ Data on cGvHD, TRM, relapse, and survival need longer follow-up. Taken together, these results confirmed that mini-dose MTX combined with standard-dose MP is safe and well tolerated.

In conclusion, the current study suggested that mini-dose MTX combined with standard-dose MP could potentially become a novel first-line therapy for patients with aGvHD. To our knowledge, these are the first integrated data from a Phase 2 clinical trial and humanized xenogeneic murine models of aGvHD. This study has provided novel evidence for the synergistic effect of GCs and MTX and a rationale for future prospective randomized clinical trials to validate our preliminary findings.
